# Supplementary material for: Timing of Chromosome DNA Integration throughout the Yeast Cell Cycle
Source: Biomolecules. 2023 Mar 29;13(4):614. doi: 10.3390/biom13040614 (PMC10135612; doi:10.3390/biom13040614)
Supplement: Supplementary file 1 [file biomolecules-13-00614-s001.zip › biomolecules-2190471 - Supplementary-revised.pdf]

biomolecules

MDPI

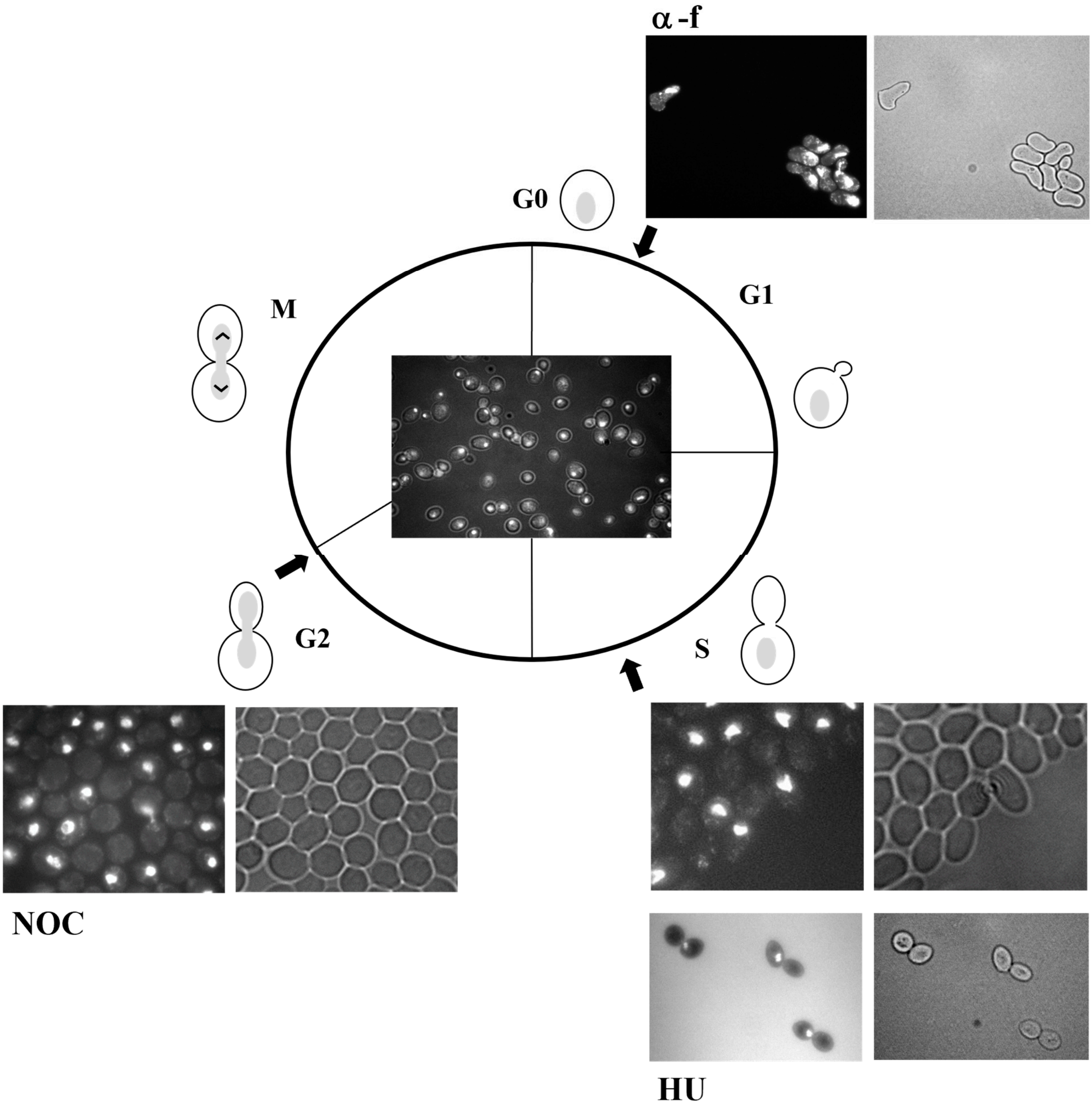

Figure S1. Arrest points of the *S. cerevisiae* mitotic cell cycle**.** Main cell cycle phases of the budding yeast on which the black arrows indicate the point within G1, S and G2/M where yeast strain San1 cells were blocked before transformation either with the BIT or SSI cassettes. a-f (a -factor), HU (hydroxyurea) and NOC (nocodazole) are the three drugs used to synchronize the *S. cerevisiae* cells. Fluorescence microscope images show cell and nuclear morphology in a San1 asynchronous cell population (center) and, clockwise from top, G1-arrested San1VTDMAT cells, late S-phase and G2/M San1 blocked cells. DAPI was used to visualize the nuclei. For HU specific single cells were also shown.

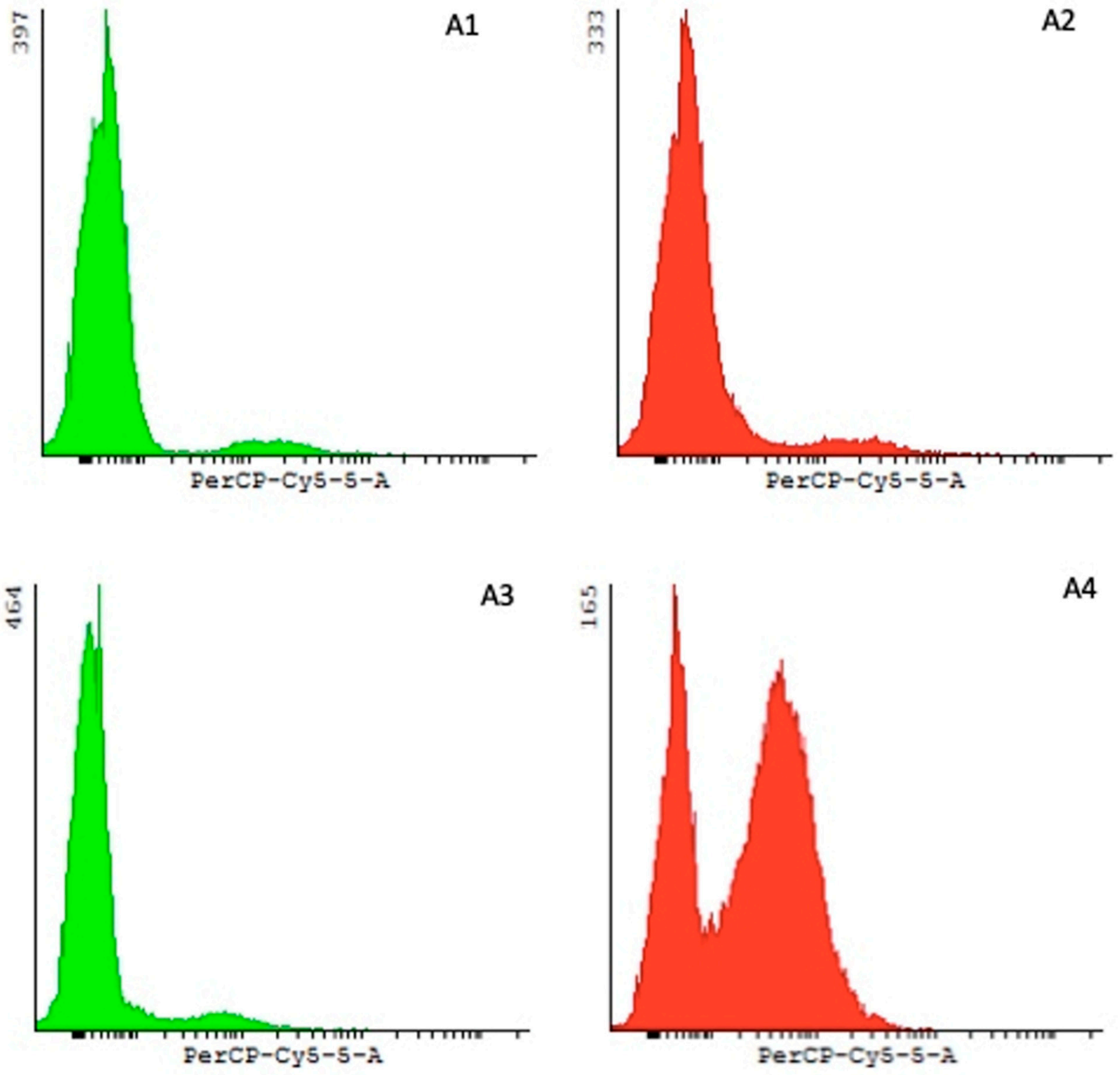

Figure S2. Flow Cytometry of translocant Susu5 compared to San1. Panel (**A1**): PI stained San1 at 7 days of ageing. Panel (**A2**): DHE stained San1 at 7 days of ageing. Panel (**A3**): PI stained Susu5 at 7 days of ageing. Panel (**A4**): DHE stained Susu5 at 7 days of ageing. All the experiments were performed three times with similar results.

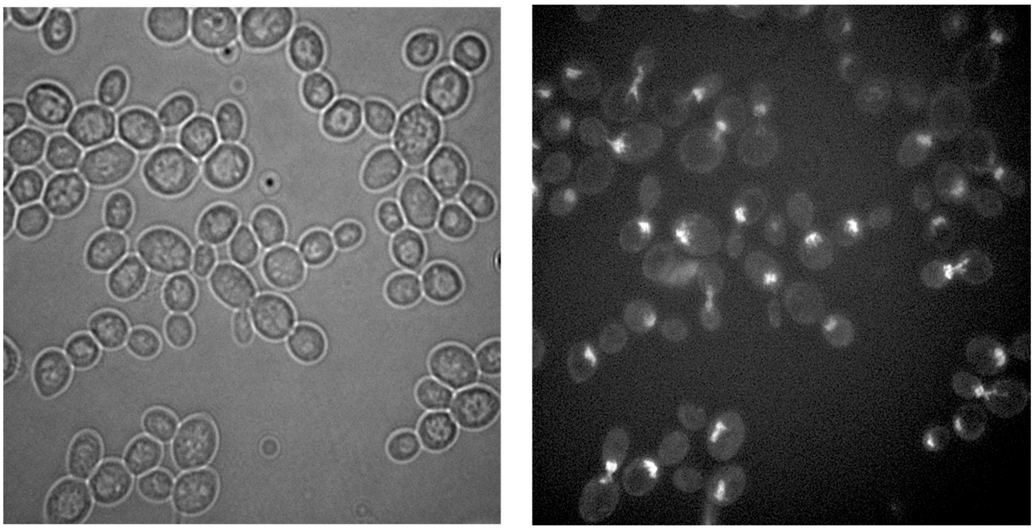

Figure S3. The double deletion of *POL32* in San1 generates a mutant showing an abnormal phenotype with an evident G2/M arrest before (left) and after (right) DAPI staining.

Table S1. The following primers were used for the construction of the BIT and SSI cassettes (lower-case letters indicate the homology with kanamycin resistance gene Kan^R) and for verification of the correct integration of the cassette; the coordinates are indicated in parenthesis and the exact location of the primers is shown in Figures 2 and 3.

**Primers used in this work**	
Translocation AD and SSI (XV-VIII):	
XVFw (ChromXV, 160604-160667)	CTTTCTTCCTTGTTTCTTTTTCTGCACAATATTTCAAGCT TACCAAGCATACAATCAACTATCgtcgacggatccccgggttaa
VIIIRev (ChromVIII, 74204-74268)	TACCCAGCGCCTTGAGGTAGCGGAGGTTTAAATTCTCC CATACTATAACAATGACAAATTTATGTccgcgcgttggccgat at
XVRev (ChromXV, 159373-159432)	GGTCAATAAGAGCGACCTCATGCTATACCTGAGAAAC AACCTGACCTACAGGAAAGAGTccgcgcgttggccgattcat
VIIIFw (ChromVIII, 74204-74271)	ATAACATAAATTTGTCATTGTTATAGTATGGGAGAATT AAACCTCCGCTACCTCAAGGCGCTGGGTAgtcgacggatcc gggttaa
VIIIRevII (ChromVIII, 72010-72076)	AGTGGATAGAATATTATATTTGAAAGCTAAATTATTT GTCAACTTGTCCGAAATGTGATGATTGTccgcgcgttggccga cat
AdhF1 (ChromXV, 159807- 159827)	TTAGCTCTAACGTATCTGGTA
AdhR1 (ChromXV, 160780-160799)	AATGAGCAACGGTATACGGC
AdhF2 (ChromXV, 159139-139158)	GTCTTGAAGCTCAGGTAAGG
AdhR2 (ChromXV, 159631-159651)	AGTCTCCAATCAAGGTTGTCG
DurF1 (ChromVIII, 74335-74354)	AGCAGTAGTATAGCCGTAGC
DurR1 (ChromVIII, 73945-73964)	AATTTGGAAGCATGCGCCAG
DurF2 (ChromVIII, 72000-72020)	GAGGTTGATGAGTGGATAGAA
DurR2 (ChromVIII, 72156-72176)	GGGATCTATCTGGTCAAACTT
Translocation SSU and SSI (XVI-IX):	

XVIFw (ChromXVI, 375433-375497)	TCTCAGTATATTTTGCTGCTTTCCTTCATATGTATATAT<br>TCTATTTACATATTAGTTTACAGAAgtcgacggatccccgggtta
IXRev (ChromIX, 36685-36749)	CTTTGCTGGGGGAGCGAGAACTACGCTAGGACAACA<br>CTCCCATACGGTAAATGTCTTAGTATGTccgcgcgttggccga<br>cat
XVIRev (ChromXVI, 376444-376508)	ACAACGTCACCTAGAGAATAAATGTTTGGAACATTGG<br>GTTCTGATATTCATCAGCAATTATTTGccgcgcgttggccgatt<br>t
IXFW (ChromIX, 35801-35865)	TTTCTAGGTGCTAGCCAGGCTGTGAGAACGTAAAATT<br>GCCTATGTTCAACATTCTCTGCTTTTCgtcgacggatccccggg<br>aa
SsuF1: (ChromXVI, 375348-375367)	CGGAGCTTTCCATTTGGAAT
SsuR1: (ChromXVI, 375570-375589)	CGATGACGAGGTAATCGTAA
SucF1: (ChromIX, 36537-36556)	ATAGGGGCTTAGCATCCACA
SucR1: (ChromIX, 36973-36992)	CCTGGACGTGGGGTCGATTA
SsuF2: (ChromXVI, 376348-376367)	ATCGATGACGTTGACGAATT
SsuR2: (ChromXVI, 376816-376835)	GAGGAATCACCAACAATGTG
SucF2: (ChromIX, 35778-35796)	TACTAACATGGCAGCATGC
SucR2: (ChromIX, 36281-36300)	CGACGGCATTAGCAAAGCTT
Primers on kanamycin resistance gene Kan^R:	
K1	ACAATCGATAGATTGTCGCAC
K2	TCAGTCGTCACTCATGGTGAT
Primers for the deletion of the MAT locus (lower-case letters indicate the homology with Kan^R):	
**Fw-MATalpha** (III 293129-293177;199496-199544;123<br>12384)	ATATATATATATATATTCTACACAGATATATACATATT<br>GTTTTTCGGGtaggcgtatcacgaggccc
**Rev-MATalpha** (III 294498-294547;200970-201019;138<br>13859)	TGACAACATTCAGTACTCGAAAGATAAACAACCTCC<br>CACGACCACACTcatcgatgataagctgtcaaac
**F1MAT** (198547-198566)	ACAGCTGAACTATGTCTGCA
**R1MAT** (293213-293232;199580-199599;12420-12439)	CCAGAATTAGCGGACCTCTT
**F2MAT** (200821-200840;13661-13680)	AAATGCAGCACGGAATATGG
**R2MAT** (201245-201264)	CGTCCCGTATAGCCAATTCG
Primers for the construction of the mutant ΔPOL32 (=pol32Δ/pol32Δ double deletant); lower-case letters indicate the homolo<br>with Kan^R:	
**Fw1Pol32**(ChromX, 517470-510)	GATCAAAAGGCGTCATATTTTATCAATGAGAAGCTCTTC<br>Cataggcgtatcacgaggccc
**Rev1Pol32**(ChromX, 516466-505)	TTGCCTTTCTTTTGAAAAAGCTTTCCAATGTTCCTTGCTTc<br>cgatgataagctgtcaaac
**Fw2Pol32**(ChromX, 517412-450)	TTTACGGATCTAATTCACCACTTGAAGATCGGCCCATCCa<br>ggcgtatcacgaggccc
**Rev2Pol32**(ChromX, 516524-563)	TGACGGCGTTTCTTGCTTTTTGAAGACGACAATGCCCTT<br>tcgatgataagctgtcaaac

**Pol32F1** (ChromX, 517647-517667)	CGGAAGTAGTAAACGCGTATG
**Pol32F2** (ChromX, 516687-516707)	GCAAACAAAGCCTTCTGAAAC
**Pol32R1** (ChromX, 517242-517262)	ATGGGATTGAAGGCATAGATG
**Pol32R2** (ChromX, 516307-516328)	CTTCGGATGGTATATTAGGATA

Table S2. Raw data of transformations.

A) BIT

1. Distribution of the events (T:translocation; adh1 and dur3: single site integration in these loci; E: ectopic integration); after transformation with the AD cassette in different phases of the cell cycle. Asynchronous populations of San1 were used as control for S, G2/M while San1VTΔMAT was used as control for G1 phase. The cells were grown 1.4 × 10^7/ml as average value. The *p*-value was determined using the Freeman-Halton extension of the Fisher probability test (see M&M). The treated cells (with HU, NOC and α-f) were compared with the asynchronous populations of San1 and San1VTΔMAT (for α-f only). San1VTΔMAT was also compared with San1 to understand if it is necessary and therefore statistically significant to use San1VTΔMAT as reference for the G1 phase-blocked cells. The single-side integration events were considered together within the same group of events for the *p*-value calculation (see M&M).

Phase	Transformations	Transformants	T	*adh1*	*dur3*	E	*p*-value
asynchronous San1	3	51	3	21	9	18	
S phase (HU)	2	107	16	27	12	52	2.23×10^{-2}
G2/M (NOC)	3	96	0	5	1	90	4.54×10^{-14}
asynchronous San1VTΔMAT	2	29	2	4	1	22	4.81×10^{-4}
G1 (α-f)	4	78	0	21	2	55	5.38×10^{-2}

2. Distribution of the events after transformation with the SUSU cassette in different phases of the cell cycle. Asynchronous populations of San1 were used as control for S, G2/M while San1VTΔMAT was used as control for G1 phase. Cells were grown 1.4 × 10^7/ml as average value.

Phase	Transformations	Transformants	T	*ssu1*	*suc2*	E	*p*-value
asynchronous San1	7	78	15	2	52	9	
S phase (HU)	13	87	8	1	70	8	1.41×10^{-1}
G2/M (NOC)	13	57	1	0	44	12	2.84×10^{-3}
asynchronous San1VTΔMAT	2	24	3	1	5	15	3.85×10^{-6}
G1 (α-f)	17	62	2	0	32	28	2.86×10^{-2}

3. Efficiency of transformation (Et) with the two cassettes (AD and SUSU) obtained dividing the number of transformants by the total number of transformed cells and by the total amount of DNA used to transform (10 µg for AD and 20 µg for SUSU). The numbers have been then normalized for the transformability of the cells with the plasmid YCp50 in same growth and cell cycle conditions.

BIT Cassette	Et San1 asynchronous	Et San1 S phase (HU)	Et San1 G2/M phase (NOC)	Et San1VTΔMAT asynchronous	Et San1VTΔMAT G1 (α-f)
AD	5×10^{-5}	223×10^{-5}	208×10^{-5}	6.710^{-5}	45×10^{-5}
SUSU	2.3×10^{-5}	14×10^{-5}	1.4×10^{-5}	0.28×10^{-5}	0.44×10^{-5}

B) SSI

Distribution of the events after transformation with the SSI cassettes at different phases of the cell cycle. Asynchronous populations of San1 were used as control since San1VTΔMAT did not show any significant difference in SSI with respect to San1 (p value ≥ 0.5). Cells were grown up to 1.4×10^7/ml.

1. *adh1* SSI raw data

Phase	Transformations	Transformants Analized	SSI	*up*	*down*	E	*p*-value
asynchronous San1	1	40 out of 400	40	0	0	0	
S phase (HU)	1	40 out of 828	38	2	0	0	2.47×10^{-1}
G2/M (NOC)	3	40 out of 86	9	0	0	31	3.82×10^{-14}
G1 (α-f)	1	40 out of 60	39	0	0	1	4.99×10^{-1}

2. *dur3* SSI raw data

Phase	Transformations	Transformants Analized	SSI	*up*	*down*	E	*p*-value
asynchronous San1	1	40 out of 46	38	0	0	2	
S phase (HU)	2	40 out of 584	40	0	0	0	0.25×10^{-1}
G2/M (NOC)	9	40 out of 40	13	2	0	25	2.78×10^{-9}
G1 (α-f)	1	40 out of 60	18	0	0	22	5.9×10^{-7}

3. *ssu1* SSI raw data

Phase	Transformations	Transformants Analized	SSI	*up*	*down*	E	*p*-value
asynchronous San1	2	40 out of 104	37	1	2	0	
S phase (HU)	6	40 out of 121	38	0	0	2	8.68×10^{-2}
G2/M (NOC)	24	40 out of 40	30	0	0	10	1.69×10^{-4}
G1 (α-f)	1	40 out of 65	39	0	0	1	1.78×10^{-1}

4. *suc2* SSI raw data

Phase	Transformations	Transformants Analized	SSI	*up*	*down*	E	*p*-value
asynchronous San1	2	40 out of 238	35	3	2	0	
S phase (HU)	2	40 out of 87	29	7	0	4	9.17×10^{-2}
G2/M (NOC)	12	40 out of 60	23	1	1	15	7.01×10^{-6}
G1 (α-f)	1	40 out of 90	38	0	0	2	1.94×10^{-2}

5. Efficiency (Et) of transformation with the SSI cassettes obtained dividing the number of transformants (above) by the total number of transformed cells and by the total amount of DNA used to transform (10 µg). The numbers have been normalized finally for the transformability of the cells with the plasmid YCp50 in same growth and cell cycle conditions. The transformability values in G1 were adjusted considering the transformability of the strain San1VTΔMAT with the plasmid YCp50.

SSI Cassette	Et asynchronous	Et S phase (HU)	Et G2/M phase (NOC)	Et G1 (α-f)
adh1	124×10^{-5}	3600×10^{-5}	195×10^{-5}	122×10^{-5}
dur3	14.3×10^{-5}	1270×10^{-5}	30.2×10^{-5}	123×10^{-5}
ssu1	16.1×10^{-5}	88×10^{-5}	10.9×10^{-5}	133×10^{-5}
suc2	37×10^{-5}	189×10^{-5}	13×10^{-5}	185×10^{-5}

C) Comparison between raw transformation data with AD cassette of the wild type strain San1 and of the mutant Δ*POL32* with and without HU. Cells were grown between 1.2-1.5 × 10^7/ml. Strain Δ*POL32* was also compared with San1 to determine the statistical significance of differences in distribution events within the two strains.

STRAIN	Transformations	Transformants	T%	*adh1*%	*dur3*%	E%	*p*-value
San1 YPD	3	51	5.9	41.2	17.6	35.3	
San1 HU	2	107	15	25.2	11.2	48.6	2.23×10^{-2}
Δ*POL32* YPD	28	48	18.7	12.5	4.2	64.6	4.24×10^{-5}
Δ*POL32* HU	3	68	0	100	0	0	1.71×10^{-23}
